# Status of insecticide resistance and its biochemical and molecular mechanisms in *Anopheles stephensi* (Diptera: Culicidae) from Afghanistan

**DOI:** 10.1186/s12936-019-2884-x

**Published:** 2019-07-26

**Authors:** Noor Halim Zahid Safi, Abdul Ali Ahmadi, Sami Nahzat, Supriya Warusavithana, Naimullah Safi, Reza Valadan, Atie Shemshadian, Marzieh Sharifi, Ahmadali Enayati, Janet Hemingway

**Affiliations:** 1 National Malaria and Leishmania Control Programme, Ministry of Public Health, Kabul, Afghanistan; 2 World Health Organization, Kabul, Afghanistan; 3 Department of Immunology, Faculty of Medicine, Mazandaran University of Medical Sciences, Sari, Iran; 4 Molecular and Cell Biology Research Center (MCBRC), Faculty of Medicine, Mazandaran University of Medical Sciences, Sari, Iran; 5 Department of Medical Entomology and Vector Control, School of Public Health and Health Sciences Research Centre, Mazandaran University of Medical Sciences, Sari, Iran; 6 Liverpool School of Tropical Medicine, Liverpool, UK

**Keywords:** *Anopheles stephensi*, Insecticide resistance, Metabolism, *kdr*

## Abstract

**Background:**

Insecticide resistance of *Anopheles stephensi*, the main malaria vector in eastern Afghanistan, has been reported previously. This study describes the biochemical and molecular mechanisms of resistance to facilitate effective vector control and insecticide resistance management.

**Methods:**

Mosquito larvae were collected from the provinces of Kunar, Laghman and Nangarhar from 2014 to 2017. The susceptibility of the reared 3–4 days old adults was tested with deltamethrin 0.05%, bendiocarb 0.1%, malathion 5%, permethrin 0.75% and DDT 4%. Cytochrome P450 content and general esterase, glutathione *S*-transferase (GST) and acetylcholinesterase (AChE) activities were measured in the three field populations and the results were compared with those of the laboratory susceptible *An. stephensi* Beech strain. Two separate allele-specific PCR assays were used to identify L1014, L1014F and L1014S mutations in the voltage gated sodium channel gene of *An. stephensi*. Probit analysis, ANOVA and Hardy–Weinberg equilibrium were used to analyse bioassay, biochemical assay and gene frequency data respectively.

**Results:**

The population of *An. stephensi* from Kunar was susceptible to bendiocarb, apart from this, all populations were resistant to all the other insecticides tested. The differences between all values for cytochrome P450s, general esterases, GSTs and AChE inhibition rates in the Kunar, Laghman and Nangarhar populations were statistically significant when compared to the Beech strain, excluding GST activities between Kunar and Beech due to the high standard deviation in Kunar. The three different sodium channel alleles [L1014 (wild type), L1014F (*kdr west*) and L1014S (*kdr east*)] were all segregated in the Afghan populations. The frequencies of *kdr east* mutation were 22.9%, 32.7% and 35% in Kunar, Laghman and Nangarhar populations respectively. *Kdr west* was at the lowest frequency of 4.44%.

**Conclusions:**

Resistance to different groups of insecticides in the field populations of *An. stephensi* from Kunar, Laghman and Nangarhar Provinces of Afghanistan is caused by a range of metabolic and site insensitivity mechanisms, including esterases, cytochrome P450s and GSTs combined with AChE and sodium channel target site insensitivity. The intensity and frequency of these mechanisms are increasing in these populations, calling for urgent reorientation of vector control programmes and implementation of insecticide resistance management strategies.

## Background

Malaria is a major endemic vector borne disease in Afghanistan. The 2018 World Malaria Report states that 27, 50 and 23% of the total population of 35,530,083 Afghans are at high, low and no risk of malaria, respectively [1]. Although there are 6 potential malaria vector species, *Anopheles stephensi* is the major vector in the eastern provinces of Kunar, Laghman and Nangarhar [2–4]. This species is common in the Middle East and the Indian subcontinent extending to South China and Myanmar [5].

In Afghanistan, the malaria control is reliant on indoor residual spraying (IRS) with deltamethrin, or more recently bendiocarb, combined with distribution of LNs mostly PermaNet [6, 7]. During 2007–2016 over 2 million deltamethrin-treated LNs were distributed in Kunar, Laghman and Nangarhar [6], with a top up distribution of 45,000 LNs in Kunar and Laghman Provinces in 2017 [6].

Insecticide resistance in *An. stephensi* to all four classes of insecticides including DDT, malathion, bendiocarb, permethrin and deltamethrin in Kunar, Laghman and Nangarhar Provinces has been reported, although it remained susceptible to bendiocarb until 2014 in Afghanistan [8, 9]. Resistance to DDT, dieldrin, malathion and more recently pyrethroids has also been reported in *An. stephensi* from the Middle East and the Indian subcontinent [6, 9–16]. Selection of resistance to bendiocarb in *An. stephensi* was recently reported from Afghanistan [6].

Several mechanisms, including metabolic resistance and site insensitivity can cause insecticide resistance [12, 15, 17–27]. In a previous study on *An. stephensi* from Afghanistan, general esterases (GES), glutathione *S*-transferases (GSTs), cytochrome P450s and insensitive acetylcholinesterase (iAChEs) were implicated in insecticide resistance [28]. Pyrethroid insecticide resistance in *An. stephensi* from India and Iran was associated with increased activity of GES and GSTs [12, 17, 20, 21, 29]. Involvement of GSTs in insecticide resistance is evident in many insects, including mosquitoes [17, 18, 24].

Knockdown resistance (*kdr*) mutation is widespread in *Anopheles* species in Africa especially *Anopheles gambiae* [30–35]. Originally, the L1014F mutation (later known as *kdr west*) was detected. In 2000, a second *kdr* mutation (*kdr east*) was detected in Kenyan *An. gambiae* [36], subsequently both mutations have been detected in other *Anopheles* conferring varying degrees of phenotypic pyrethroid resistance [5, 10, 37–43].

The first report of a *kdr* L1014F resistance mechanism in *An. stephensi* was in the DUB-S strain in 2003 [19]. Recently, *kdr east* and *kdr west* mutations have been detected in *An. stephensi* from India [5]. In 2014, target site insensitivity for pyrethroid insecticides was studied in *An. stephensi* from Kunar and Nangarhar Provinces of Afghanistan [43]. The wild type susceptible L1014 allele in the sodium channel gene was the most prevalent followed by L1014S (*kdr east*, 21.4%) and L1014F (*kdr west*, 1.4%), no *kdr* homozygotes were collected.

The World Health Organization (WHO) recommends that insecticide susceptibility status of malaria vectors should be monitored annually [44, 45]. When insecticide resistance is detected, its intensity and the biochemical and molecular mechanisms should also be investigated [44, 45]. Accurate information on the underlying resistance mechanisms and their intensity or frequency in malaria vectors can then inform vector control programmes and ensure timely management of insecticide resistance. Following the WHO Global Plan for Insecticide Resistance Management (GPIRM) [46], in this study the insecticide resistance status and its underlying mechanisms were investigated in *An. stephensi* from Kunar, Laghman and Nangarhar Provinces in eastern Afghanistan.

## Methods

### Study area

**Fig. 1:**
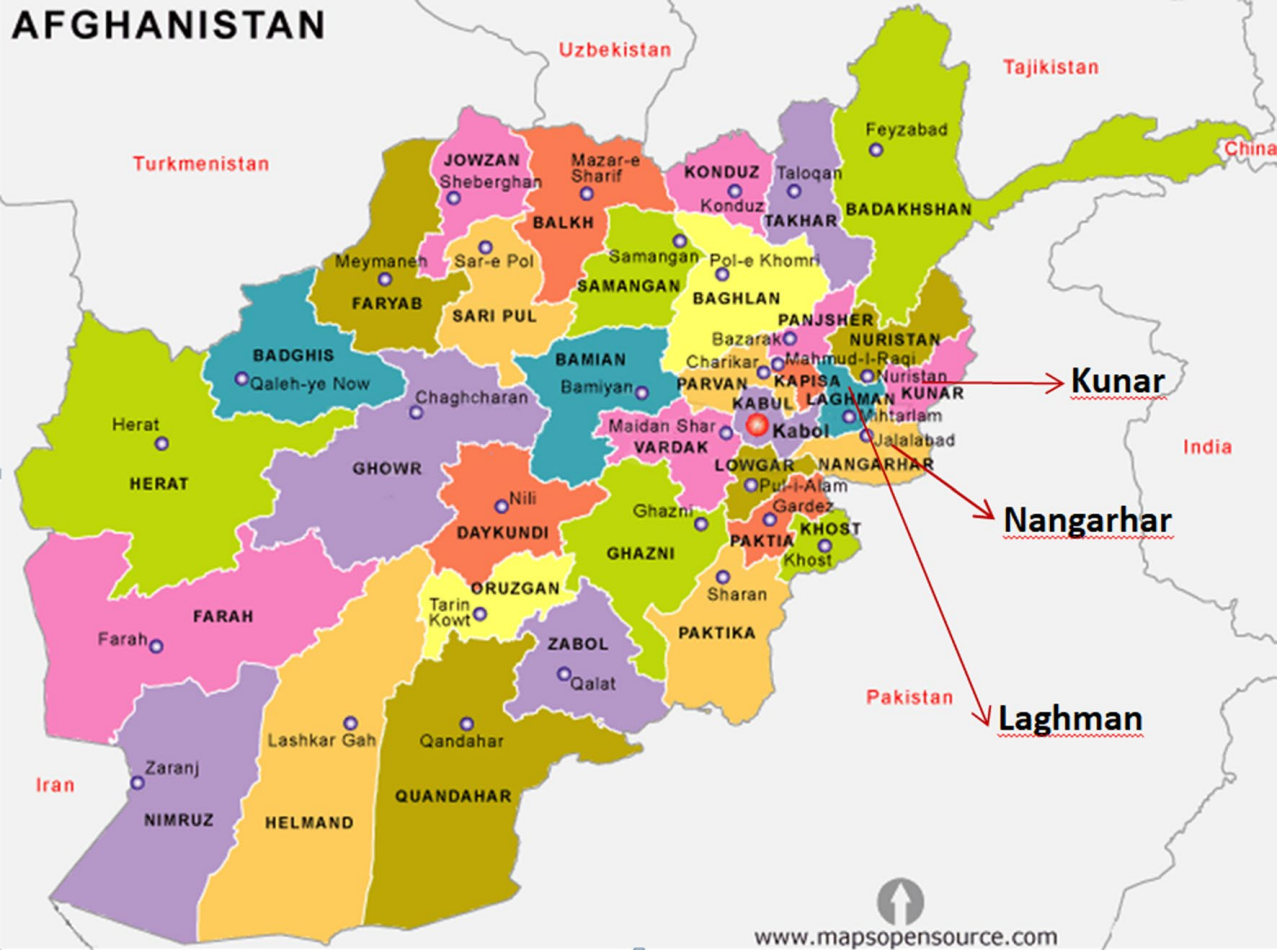
Map of Afghanistan and the location of the provinces of Kunar, Nangarhar and Laghman

The study areas were the provinces of Kunar (34.8466°N, 71.0973°E), Laghman (34.6898°N, 70.1456°E) and Nangarhar (34.1718°N, 70.6217°E) in East Afghanistan (Fig. 1), sample site details are given in Table 1.

**Table 1:** Localization of sample collection sites and habitat description

Province	District	Village	Habitat type	Elevation (m)	Altitude	Longitude
Kunar	Nurgal	Nurgal	River stream	658	34°36′45.70″N	70°46′31.76″E
	Chawkay	Babur	Pond, river stream	711	34°41′26.04″N	67°30′42.61″E
Laghman	Mihtarlam	Tirgari	River stream	735	34°38′41.03″N	70°12′36.20″E
		Qarghayi	River stream	644	34°32′53.46″N	70°14′29.18″E
	Qarghayi	Swati	River stream	635	34°38′41.03″N	70°12′36.20″E
Nangarhar	Behsood	Bagrami	Pond/river stream	571	34°26′49.08″N	70°24′24.94″E
		Saracha	River streams	540	34°23′13.70″N	70°32′23.52″E
	Samar Khel	Gujranu Bella	Swamp, river	525	34°22′39.58″N	70°34′50.33″E

### Larval collection and mosquito rearing

Thousands of larvae were collected from multiple breeding sites in the provinces of Kunar, Laghman and Nangarhar from 2014 to 2017. Breeding sites with the highest larval density were used for sampling to obtain enough specimens for susceptibility tests and biochemical and molecular analyses.

Field collected larvae were reared to the adult at 25 ± 2 °C temperature and 75 ± 10% relative humidity. The adults were identified to species using Glick’s identification keys [47]. Sugar-fed 3–4 days old adult *An. stephensi* mosquito specimens were used for bioassays and biochemical and molecular analyses. The susceptible Beech strain of *An. stephensi* was provided by the Department of Medical Entomology, School of Public Health, Tehran University of Medical Sciences, Iran.

### Insecticide susceptibility tests

Insecticide susceptibility tests were carried out according to the standard WHO procedure for mosquito adults [45]. The WHO supplied insecticide-impregnated papers of DDT 4%, malathion 5%, bendiocarb 0.1%, permethrin 0.75% and deltamethrin 0.05%, were used for bioassays. Mosquitoes were divided into batches of 25 before being exposed to the insecticide-treated papers for 1 h. Experiments were conducted under insectary conditions with a minimum of four replicates per bioassay. For control replicates, silicone-treated papers were used. The results of the bioassay were discarded if the mortality of the control replicates was over 20%. Abbott’s formula was used to correct the mortality if it was between 5 and 20% [45].

### Biochemical assays

The biochemical assays were performed according to the protocol of WHO/WHOPES [48]. The enzyme activity of GSTs and GES as well as the cytochrome P450s content and inhibition rates of AChE (using propoxur) were measured.

### Molecular methods

#### DNA isolation

DNA from individual mosquitoes was extracted using the Livak buffer extraction method [49] with some modifications. Livak buffer contained 80 mM NaCl, 1.57% Tris Base, 0.5% sodium dodecyl sulfate (SDS), 5.5% sucrose and 50 mM EDTA. Individual mosquitoes were homogenized in 100 µl pre-heated (65 °C) Livak buffer in 1.5 ml Eppendorf tubes using a plastic pestle. Homogenates were incubated at 65 °C for 30 min. Potassium acetate was added to each tube to a final concentration of 1 M before incubating the mixture on ice for 30 min. The tubes were centrifuged at 12,000*g* for 15 min at 4 °C. Supernatants were transferred to clean tubes and mixed with 200 µl ice-cold ethanol, followed by centrifugation at 12,000*g* for 15 min at 4 °C. Pellets were rinsed in 100 µl 70% ice-cold ethanol, spun at 12,000*g* for 5 min at 4 °C and re-suspended in 50 µl pre-heated Tris–EDTA (TE) buffer or nuclease free water.

#### Primer design and PCR

**Fig. 2:**
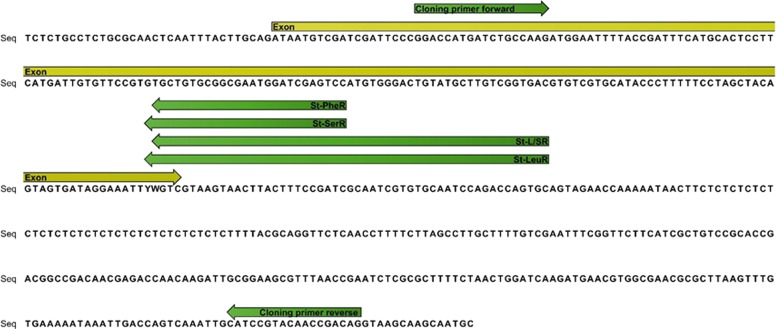
Schematic diagram of a IIS6 fragment of vgsc gene where the primers for cloning and screening sit

To obtain a fragment of voltage gated sodium channel (vgsc) encompassing the *kdr* locus, a DNA section from IIS6 segment of *An. stephensi* was amplified using primers *kdr*F (5′-GGA CCA YGA TTT GCC AAG AT-3′) and *kdr*R (5′-TGG TGC AGA CAA GGA TGA AG-3′) in a reaction mixture (25 μl) that contained 1× buffer, 1.5 mM of MgCl2, 200 μM of each dNTP, 0.5 μM of each primer and 0.625 unit of Taq DNA polymerase. The conditions of PCR were: an initial denaturation at 95 °C for 5 min, followed by 35 cycles at 95 °C for 30 S, 48 °C for 30 S and 72 °C for 45 S, and a final extension step at 72 °C for 7 min. The PCR products were purified with Takapozist PCR purification kit (Takapozist, Iran) and were sequenced in both directions by Macrogen Inc, Korea using BigDye (Applied Biosystem Chemistry). A schematic diagram showing the stretch of the IIS6 of the sodium channel gene where the primers for cloning and screening sit is given in Fig. 2.

#### Positive controls and cloning

Positive control for *kdr east* mutation (L1014S) was the courtesy of Dr. OM Singh, National Malaria Research Center, India. Positive control for the *kdr west* mutation (L1014F) was from initial PCR screening of some specimens that survived bioassay using *kdr*F and *kdr*R primers. Wild type positive control (L1014) was the product of PCR of two specimens from the susceptible Beech strain of *An. stephensi* with aforementioned primers. All positive control fragments were confirmed by sequencing and then cloned in pTG19-T system (Sinaclon, Iran) according to the manufacturer’s instruction and used as positive control in genotyping reactions.

#### Genotyping *kdr* alleles

The screening method developed by Singh et al. was used in this study with some modifications [5]. Two PCR reactions were carried out for each DNA sample from individual mosquito specimens. In both PCRs, the forward primer used was *kdr*F. In the first PCR (hereafter called PCR-1), the allele 1014F is discriminated from the wild type (L1014) and in the second PCR (hereafter called PCR-2), 1014S and wild type alleles are discriminated. The allele-specific primers were the same used by Singh et al. [5]. They are St-L/SR (5′-GCG GGC AGG GCG GCG GGG GCG GGG CCC GAT CGG AAA GTA AGT TAC TTA CGt CT-3′) and St-PheR (5′-GAT CGG AAA GTA AGT TAC TTA CGg CA-3′) for PCR-1, and St-LeuR (5′-GCG GGC AGG GCG GCG GGG GCG GGG CCC GAT CGG AAA GTA AGT TAC TTA CGA gTA-3′) and St-SerR (5′-CGA TCG GAA AGT AAG TTA CTT ACG AtT G-3′) for PCR-2. The expected amplicon size formed by the allele-specific primers St-L/SR and St-PheR (with primer *kdr*F) in PCR-1 are 218 and 191 bp receptively. The expected size of amplicons in PCR-2 with allele specific primers St-LeuR and St-SerR are 218 and 192 bp, respectively.

#### Data management, transformation and analyses

The reading of the activity/content of the enzymes were done in a UV/visible microtitre plate reader (BioTek, USA) run under KC junior software and the data were directly extracted to Microsoft Excel for further analysis. Mean values of activity or contents of each enzyme of all populations were compared by ANOVA in conjunction with the Tukey’s statistical test using SPSS version 19 software. Enzyme ratios (ER) were calculated by dividing the mean activities or content of the enzymes in the field populations with those of the Beech susceptible strain.

The molecular analyses data were calculated based on the frequency of *kdr* mutations in *An. stephensi* populations from different provinces in dead or alive specimens following bioassays. Hardy Weinberg equilibrium software was used for analysis between different *kdr* genotypes [50].

## Results

### Mosquito samples

Approximately 2250, 1800 and 2250 3–4 days old adult mosquitoes were used for bioassays in 2014, 2015 and 2017, respectively. Two hundred 3–4 days old adult mosquito specimens were used for biochemical assays. Dead and alive adult mosquitoes following bioassays with deltamethrin from each population were used for molecular analysis.

### Bioassay

Considering the susceptibility threshold of a mortality rate above 98%, then only one population of *An. stephensi* from Kunar was susceptible to bendiocarb and the Laghman and Nangarhar populations were resistant to all insecticides tested. When bioassay mortalities in 2014 were compared to those of 2017, there was a reduction of 27% in susceptibility to permethrin in Kunar population, while the susceptibility to permethrin in the other two field populations remained more or less the same. Susceptibility to deltamethrin was the same in 2014 and 2017 in Kunar population, whereas an increase of about 30% in susceptibility to deltamethrin was observed in the other two field populations. Regarding susceptibility to malathions, reduction of 55%, 46% and 41% were monitored in the Kunar, Laghman and Nangarhar populations, respectively. The susceptibility to bendiocarb increased 3% in the Kunar population, changing the status of this population from being resistant to susceptible. The susceptibility to bendiocarb in the other two field populations decreased 43.5% and 16%. Surprisingly, the susceptibility to DDT increased 55% in the Kunar population, however, in Laghman population it reduced about 44% while it remained the same in the Nangarhar population (Table 2).

**Table 2:** Percentage mortality of bioassays on *An. stephensi* adults using different insecticides in 2014 to 2017

Insecticide	Province	2014	2015	2017	Percentage changes in the susceptibility compared to the 2014 baseline
Permethrin 0.75%	Kunar	89	89	65	− 27
	Laghman	90	87	91	+ 1
	Nangarhar	92	92.2	90	− 2
Deltamethrin 0.05%	Kunar	78	85	78	0
	Laghman	65	63	93	43
	Nangarhar	66	95	90	36
Malathion 5%	Kunar	62	88	28	− 55
	Laghman	95	85	51	− 46
	Nangarhar	95	86	56	− 41
Bendiocarb 0.1%	Kunar	95	90	98	+ 3
	Laghman	92	63	52	− 43.5
	Nangarhar	100	86	84	− 16
DDT 4%	Kunar	45	NA	70	+ 55.5
	Laghman	80	NA	45	− 44
	Nangarhar	60	NA	60	0

### Esterase activity

The esterase activity ratios of *An. stephensi* from Kunar, Laghman and Nangarhar Provinces in East Afghanistan compared to the susceptible Beech strain are given in Table 3. The mean activity of alpha- and beta-naphthyl acetate were 0.000983 and 0.000961 µM/min/mg protein in the Kunar population, 0.001 and 0.001 µM/min/mg protein in the Laghman population and 0.000911 and 0.000904 µM/min/mg protein in the Nangarhar population and 0.000517 and 0.000567 µM/min/mg protein in the susceptible Beech strain. The esterase activities of mosquitoes from the Kunar, Laghman and Nangarhar populations were statistically significantly higher than those of the susceptible Beech strain at the 5% level. The esterase activity in the Laghman population was marginally higher than those of the Kunar and Nangarhar populations (Table 3).

**Table 3:** Mean enzyme activities and enzyme ratios (ER) measured in *An. stephensi* populations from Afghanistan

Enzyme	Population	Mean	ER
Alfa esterase	Susceptible	0.000517	1
	Kunar	0.000983	1.90
	Laghman	0.001050	2.03
	Nangarhar	0.000917	1.77
Beta esterase	Susceptible	0.000567	1
	Kunar	0.000961	1.69
	Laghman	0.001075	1.89
	Nangarhar	0.000904	1.59
GST	Susceptible	0.11445	1
	Kunar	0.16204	1.41
	Laghman	0.23021	2.01
	Nangarhar	0.21822	1.90
cytochrome p450	Susceptible	0.0000491	1
	Kunar	0.0001316	2.68
	Laghman	0.0001167	2.37
	Nangarhar	0.0001204	2.45
% AChE inhibition	Susceptible	68.63	1
	Kunar	51.98	0.75
	Laghman	48.82	0.71
	Nangarhar	53.14	0.77

ERs are the results of the mean enzyme activity or content of the field populations divided by those of the susceptible Beech population. %AChE inhibition is the percentage of acetylcholinesterase inhibition of the field populations compared with the susceptible Beech population

### GSTs activity

The activity of the GSTs in the Laghman and Nangarhar populations were significantly higher than that of the susceptible Beech strain, however, the differences between the GST activity of Kunar population was not significantly different from that of the susceptible strain (Table 3).

### Cytochrome P450s contents

The ratio of cytochrome P450s in the Kunar, Laghman and Nangarhar populations were 2.68, 2.37 and 2.45 when compared with that of the susceptible Beech strain (Table 3). However, the differences of the cytochrome P450 contents between the Kunar, Laghman and Nangarhar populations were not statistically significant (Table 3).

### AChE inhibition

The AChE inhibition rates with the carbamate propoxur were 68.63% in the susceptible Beech strain, 51.98% in the Kunar population, 48.82% in the Laghman population and 53.14% in the Nangarhar population (Table 3). The inhibition levels in all field populations were lower than the threshold of 60% set for considering the AChE insensitive to propoxur. There were significant differences between the three populations in AChE inhibition when compared with that of the susceptible Beech strain (p > 0.001). However, the differences between the inhibition rates of AChE in the field populations were not statistically significant from each other (p = 0.09). The frequency of individuals with iAChE (inhibition less than 60% with propoxur) were 37%, 64%, 78% and 75% in susceptible, Kunar, Laghman and Nangarhar populations of *An. stephensi*, respectively.

In summary, the differences between the activities/contents of all the enzymes measured in this study in the Kunar, Laghman and Nangarhar populations were statistically significant compared with those of the susceptible Beech strain. The Laghman population had a marginally higher enzymes activities/content compared with the other Afghan field populations.

### *kdr* genotyping

Primers designed to amplify a flanking region encompassing the SII6 region containing the *kdr* codon of the vgsc of *An. stephensi* gave a 493 bp product, the results of sequencing this region are shown in Fig. 2. The cloned products of the *kdr* alleles were used as positive control in genotyping reactions.

**Fig. 3:**
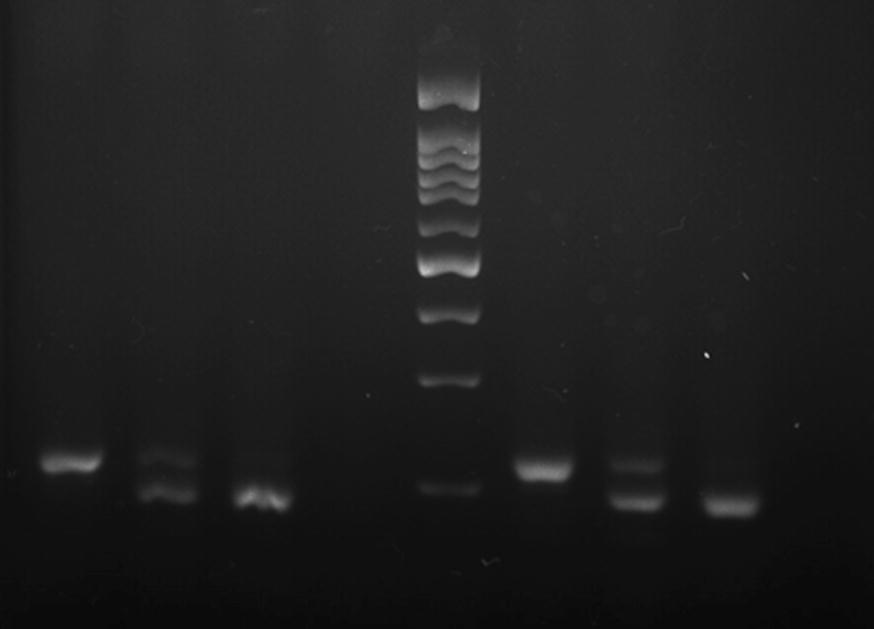
Banding pattern of the kdr genotyping of *An. stephensi* specimens from Kunar, Laghman and Nangarhar populations from Afghanistan. Lanes 1: LL, 2: L/F, 3: F/F, 4: negative control, 5: 100 bp DNA ladder, 6: L/L, 7: L/S, 8: S/S

Using the forward primer designed in this study and four reverse primers, a total of 180 mosquitoes were successfully genotyped for all three different alleles (homozygote and heterozygote) of wild type, *kdr* west and *kdr east* (Fig. 3). The results are summarized in Table 4.

**Table 4:** The results of screening kdr mutations in the field populations of *An. stepehsni* from Kunar, Laghman and Nangarhar Provinces collected from Afghanistan in 2017

Strain	Dead/alive after bioassay	*Kdr-west*, homozygote	*Kdr-west* heterozygote	*Kdr-east* homozygote	*Kdr-east* heterozygote	Wild type homozygote	Subtotal
Laghman	Alive	1	2	1	6	12	22
	Dead	0	0	0	12	27	39
Kunar	Alive	1	1	1	4	13	20
	Dead	0	1	1	8	34	44
Nangarhar	Alive	1	1	1	8	9	20
	Dead	0	0	0	10	25	35
Subtotal		3	5	4	48	120	180

The total frequency of *kdr west* in all populations was 4.44% (5 heterozygotes and 3 homozygotes). The frequency of *kdr* in the three populations of Kunar, Laghman and Nangarhar were not significantly different. The frequency of the *kdr* alleles in pyrethroid bioassay survivors were, however, significantly different from the dead mosquitoes in all populations tested. Seven out of 8 *kdr west* mutated individuals were among bioassay survivors. Individuals homozygote for *kdr west* were detected only in the bioassay survivors (one in each population).

The frequency of *kdr east* was significantly higher than *kdr west*. The overall frequency of *kdr east* was 29%. The frequency of *kdr east* in the alive and dead specimens of the studied populations were 38% and 26.7%, respectively. The frequency of *kdr east* in Nangarhar, Laghman and Kunar populations were 35%, 32.7% and 22.9% respectively. Three of the four homozygote *kdr east* mutants were among the bioassay survivors.

Regardless of the type of the mutated alleles, the frequency of *kdr* in all populations were 33.33%. The frequency of *kdr east* and *west* combined in the populations were in the order of Nangarhar 39.6%, Laghman 37.9% and Kunar 27.9%. The allelic frequency of Leucin, Serine and Phenylalanine were 0.814, 0.156 and 0.03 respectively, all within the HWE (p = 0.14).

## Discussion

A general reduction in the susceptibility of *An. stephensi* from the eastern provinces of Afghanistan in recent years is probably in part due to the development of agriculture [51, 52] and increased use of different classes of insecticides that exerted a selection pressure on mosquitoes, especially where the larval breeding sites are within or close to the rice fields [43]. The increase in resistance was most obvious to organophosphates and carbamates. Interestingly, the susceptibility to deltamethrin increased in the Laghman and Nangarhar populations, whereas to bendiocarb, it decreased in these populations. It might be due to the recent use of bendiocarb in rotation with deltamethrin in the region. Although resistance to bendiocarb has been reported in different mosquito species [53–56], development of bendiocarb resistance in *An. stephensi* populations in Afghanistan is now a cause for concern for the malaria control programme, who use this in IRS as an alternative to deltamethrin, in combination with LNs to manage insecticide resistance [2]. Malathion and bendiocarb share the same mode of action and have the same target site resistance mechanism [57, 58]. The recent selection of bendiocarb resistance combined with the longstanding resistance to malathion [4, 9] suggests multiple resistance mechanisms in the Laghman population of *An. stephensi*.

Differential patterns of selection of insecticide resistance in populations of *An. stephensi* in different provinces of Kunar, Laghman and Nangarhar were observed [6]. For example, the Kunar population remained susceptible to bendiocarb whereas the populations in Laghman and Nangarhar developed resistance to this insecticide between 2014 and 2017. Resistance levels to the pyrethroid insecticides permethrin and deltamethrin remained low, suggesting that resistance is conferred only by the relatively weak *kdr* mechanism.

Biochemical assays suggested that multiple metabolic mechanisms of resistance may have been selected in *An. stephensi* from Afghanistan. GST-based metabolism is often associated with DDT resistance [59–61]. GSTs activities were the highest in Laghman population compared with the other two field populations, and DDT resistance levels in Laghman population were the highest among all three field populations. Besides involving in DDT resistance, GSTs may be secondarily involved in pyrethroid insecticide resistance, so care must be taken in interpretation of the dynamics of this enzyme group as its metabolic role is not restricted to DDT [62].

Cytochrome P450s content in the Kunar, Nangarhar and Laghman populations of *An. stephensi* is increasing. Implications of this information for looking at the potential for moving to the new generations of PBO LNs should be considered [63]. There are evidence that PBO LNs improved control of malaria transmission compared with standard long-lasting insecticidal nets where pyrethroid resistance is prevalent [64].

The mean inhibition rates and frequency of iAChE individuals in the field populations of *An. stephensi* from Afghanistan are significantly higher than that of the susceptible Beech strain. The iAChE should confer resistance to malathion and bendiocarb in *An. stephensi* [65, 66].

The importance of different enzyme groups in conferring insecticide resistance in different insects, especially mosquitoes, is overwhelming [12, 21, 23, 24, 27, 29, 55]. General esterases and cytochrome P450s are involved in pyrethroid insecticide resistance in *An. stephensi* from Dubai, Iran and India [12, 20, 21, 23, 67]. Esterases also confer resistance to OPs and to a lesser extent to pyrethroid insecticides [19, 68]. Involvement of iAChE in OPs and carbamate insecticides resistance is evident in many insect groups including *An. stephensi* from Iran, Afghanistan and India [12, 23, 27, 28]. A similar pattern of AChE insensitivity was seen in *Anopheles albimanus* in Mexico [69], in Turkish populations of the *Anopheles maculipennis* [70], and *An. stephensi* from Iran [13]. The reduction in susceptibility of *An. stephensi* from Afghanistan to bendiocarb in recent years [6] is of concern, as it is the insecticide of choice for IRS and insecticide resistance management in the East of Afghanistan is reliant on this in combination with LN distribution [2]. Close monitoring is now required to ensure that the situation does not deteriorate and impact on the ability to control malaria.

The current study suggests that both metabolic and target site resistance have increased between 2014 and 2017 in Afghanistan. Knock down resistance mechanism of pyrethroid resistance in *An. stephensi* was first determined in 2003 by Enayati et al. [19] in the DUB-S strain. At that time, only L1014F (later known as *kdr west*) mutation was observed in the species. After the discovery of L1014S mutation (known as *kdr east*) in *An. gambiae* in Kenya [36], reports of the presence of this trait in different mosquito species emerged [37, 38, 40, 71]. The presence of *kdr east* in *An. stephensi* from India was reported for the first time by Singh et al. [5]. The development of *kdr* mechanism in *An. stephensi* from Afghanistan was first reported in 2016 [43], a report that triggered this bigger study in populations of *An. stephensi* from Kunar, Laghman and Nangarhar.

The overall frequency of *kdr east* in Afghan *An. stephensi* has increased by 8% between 2016 and 2017 [43]. The *kdr* mutants were significantly more frequent in bioassay survivors compared to dead (38% vs 27%), as expected from this relatively weak pyrethroid resistance mechanism. The allelic frequency of *kdr east* was much lower than that reported in an Indian population of *An. stephensi* [5], however, the allelic frequency of *kdr west* was higher. *Kdr west* and *east* are prevalent in different *Anopheles* species worldwide [72, 73]. In *An. gambiae* in many West African countries, the frequency of *kdr west* has reached fixation (total homozygocity) [54, 74–77]. In other parts of Africa, co-occurrence of *kdr west* and *kdr east* with relatively high frequency is reported [34, 37, 71, 73, 78]. It has been argued that even with high *kdr* mutation rates, LNs may still be effective in protecting people against malaria [79–85]. However, a systematic review with meta-analysis revealed that the efficacy of the LNs diminishes with high intensity insecticide resistance [86, 87].

The development of different *kdr* traits may reflect the history of phenotypic resistance to different classes of insecticides. It was shown that *kdr west* confers stronger resistance to pyrethroid insecticides compared with *kdr east*, as the latter confers higher resistance to DDT and lesser to pyrethroids [88]. The susceptibility of Laghman population of *An. stephensi* to DDT (45%) is lower compared to Kunar (the highest 70%) and Nangarhar populations (60%).

The proportion of arable land to the total area of the country is in the order of Nangarhar (12%) > Kunar (7%) > Laghman (4%) [52]. The intensive use of organophosphorus and carbamate insecticides in agriculture, coupled with the historical and current use of these insecticides in public health has selected increasing levels of resistance to malathion and bendiocarb in Afghan *An. stephensi* between 2014 and 2017 [6]. Larviciding with temephos and IRS using bendiocarb may exacerbate the levels of resistance to these classes of insecticides in *An. stephensi*.

The results of biochemical assays support the development of resistance to all classes of insecticides in *An. stephensi* in these provinces. A detailed resistance management plan should now be prepared to address potential issues going forward before resistance problem start to reduce the effectiveness of malaria control.

## Conclusions

*Anopheles stephensi* populations from Kunar, Laghman and Nangarhar developed a range of resistance to different insecticides. Resistance to DDT, malathion, bendiocarb and pyrethroid insecticides is evident in different populations of the mosquito. Different enzyme groups are involved in the resistance to insecticides in *An. stephensi* from Kunar, Laghman and Nangarhar Provinces of Afghanistan. The contents of cytochrome P450s and the levels of activities of general esterases, glutathione *S*-transferases and the insensitivity of acetylcholinesterase are increasing in these populations. Based on the results, it can be concluded that the strength of metabolic resistance in *An. stephensi* from Laghman is slightly higher to multiple insecticides than the other two field populations. Knockdown resistance (both L1014F and L1014S) gene frequency is also on the rise. Moreover, homozygote individuals for either *kdr* traits have been detected for the first time in the field populations. These observations have huge technical as well as practical implications to malaria control programmes in Afghanistan. Based on these observations, the following general recommendations can be made: (i) vector control programmes need to be evidence-based and to be guided by routine monitoring and evaluation of vector control interventions including susceptibility to insecticides; (ii) malaria vector control is implemented based on careful stratification which includes, among other things, vector susceptibility to insecticides and their underlying resistance mechanisms; (iii) close collaboration of public health and agriculture sectors is required for effective management of insecticide resistance. In other words, for sound insecticide resistance management, implementation of strategies such as IVM in public health and IPM in agriculture is recommended. On the other hand, in planning and implementation of control measures for vector-borne diseases especially malaria, considerations should be made not only to the growing insecticide resistance status in the vectors, but also to its mechanisms in *An. stephens*i in the East of Afghanistan. Given the differential development of insecticide resistance and its underlying mechanisms in *An. stephensi* populations from different provinces of Afghanistan, it is recommended that the malaria control planning be province specific e.g. applying bendiocarb IRS in Kunar where *An. stephensi* is susceptible to this insecticide, while deploying PBO nets in Laghman and Nangarhar Provinces where cytochrome P450 is involved in pyrethroid insecticides resistance. LNs with alternative insecticides e.g. chlorfenapyr, and primiphos methyl long lasting IRS may also be answers to some of the challenges of insecticide resistance in *An. stephensi* in Afghanistan.

## Data Availability

All data generated or analysed during this study are included in this published article.

## Abbreviations

iAChE: insensitive acetylcholinesterase
AChE: acetylcholinesterase
bp: base pair
DDT: dichlorodiphenyltrichloroethane
EDTA: ethylenediaminetetraacetic acid
GPIRM: global plan for insecticide resistance management
GES: general esterase
GST: glutathione *S*-transferase
HWE: Hardy–Weinberg equilibrium
IRS: indoor residual spraying
KDR: knockdown resistance
LNs: long-lasting insecticidal nets
MoPH: Ministry of Public Health
NLMCP: National Malaria and Leishmaniasis Control Programme
OPs: organophosphates
PCR: polymerase chain reaction
P450s: cytochrome P450s
SDS: sodium dodecyl sulfate
TE: Tris–EDTA
VGSC: voltage gated sodium channel
WHO: World Health Organization
WHOPES: World Health Organization Pesticide Evaluation Scheme
